# Role for Chlamydial Inclusion Membrane Proteins in Inclusion Membrane Structure and Biogenesis

**DOI:** 10.1371/journal.pone.0063426

**Published:** 2013-05-17

**Authors:** Jeffrey Mital, Natalie J. Miller, David W. Dorward, Cheryl A. Dooley, Ted Hackstadt

**Affiliations:** 1 Host-Parasite Interactions Section, Laboratory of Intracellular Parasites, Rocky Mountain Laboratories, National Institute of Allergy and Infectious Diseases, National Institutes of Health, Hamilton, Montana, United States of America; 2 Department of Biomedical Sciences, Quinnipiac University, Hamden, Connecticut, United States of America; 3 Microscopy Unit, Research Technologies Branch, Rocky Mountain Laboratories, National Institute of Allergy and Infectious Diseases, National Institutes of Health, Hamilton, Montana, United States of America; University of California Los Angeles, United States of America

## Abstract

The chlamydial inclusion membrane is extensively modified by the insertion of type III secreted effector proteins. These inclusion membrane proteins (Incs) are exposed to the cytosol and share a common structural feature of a long, bi-lobed hydrophobic domain but little or no primary amino acid sequence similarity. Based upon secondary structural predictions, over 50 putative inclusion membrane proteins have been identified in *Chlamydia trachomatis*. Only a limited number of biological functions have been defined and these are not shared between chlamydial species. Here we have ectopically expressed several *C. trachomatis* Incs in HeLa cells and find that they induce the formation of morphologically distinct membranous vesicular compartments. Formation of these vesicles requires the bi-lobed hydrophobic domain as a minimum. No markers for various cellular organelles were observed in association with these vesicles. Lipid probes were incorporated by the Inc-induced vesicles although the lipids incorporated were dependent upon the specific Inc expressed. Co-expression of Inc pairs indicated that some colocalized in the same vesicle, others partially overlapped, and others did not associate at all. Overall, it appears that Incs may have an intrinsic ability to induce membrane formation and that individual Incs can induce membranous structures with unique properties.

## Introduction

Chlamydiae are obligate intracellular bacteria that are the etiologic agents of a variety of diseases affecting humans or animals. Human diseases include trachoma and sexually transmitted diseases caused by *Chlamydia trachomatis* [1] as well as community acquired pneumonia caused by *C. pneumoniae* [2]. *C. psittaci* is of veterinary importance and can occasionally lead to zoonotic infections [3]. A number of other *Chlamydia* species are restricted to specific animal species. Examples include *C. muridarum*, *C. caviae,* and *C. felis*, which are generally limited to mice, Guinea pigs, and cats, respectively [4]–[6].

Despite the great diversity in disease, tissue tropism, and species specificity, the sequenced genomes of chlamydiae display a very high degree of synteny and sequence similarity. The limited number of genes that have been associated with pathobiotype is considered a paradox of chlamydial biology [7]–[15]. At a cellular level, however, the interactions of the different chlamydial species are very similar [16]. All chlamydiae share a simple developmental cycle that consists of cell types adapted for extracellular survival, the elementary body (EB), or for intracellular replication, the reticulate body (RB) [17]. The chlamydial developmental cycle takes place within a vacuole, called an inclusion, unlike that of any other known intracellular pathogens [18]–[20]. The chlamydial inclusion is non-fusogenic with any known components of the endocytic pathway but instead appears to intersect an exocytic pathway from which it intercepts the eukaryotic lipids sphingomyelin and cholesterol for incorporation into their cell walls. The mechanistic details of this pathway are largely unknown [21] but are clearly defined by the parasite as chlamydial transcription and translation is required for initiation of the process [22].

Within a few hours of parasite mediated endocytosis, EBs initiate de novo transcription and translation. Among the early events of chlamydial infection is extensive modification of the inclusion membrane by the insertion of a number of type III secreted effector proteins [23]. The inclusion membrane proteins (Incs) are characterized by a bi-lobed hydrophobic domain of 40 amino acids or more. Despite this secondary structural feature, they share little sequence similarity with known proteins or each other [24]. *C. trachomatis* has been predicted to encode from 39 to 59 putative Incs. Of those, approximately half have now been confirmed by localization in the inclusion membrane [11], [23], [25]–[28]. Although the Incs are exposed on the cytosolic face of the inclusion membrane and situated such that they would be positioned to control genus-specific interactions with the host cell, there is a notable diversity in Inc proteins encoded by the different chlamydial species. Moreover, chlamydial species appear to encode their own complement of Incs. In one study, orthologs of 27 out of 55 putative *C. trachomatis* Incs were not detected in *C. pneumoniae* [29]. Conversely, *C. pneumoniae* was predicted to encode 92 putative Incs, most of which were not found in *C. trachomatis* [26], [28], [29]. Thus Inc content in different chlamydial species is remarkably divergent.

Here we have examined ectopic Inc expression in HeLa cells and describe novel membranous structures induced by Inc expression. Five Incs were expessed as mCherry or GFP fusions and formed vesicular structures in the cytosol. No endocytic or cellular markers were associated with these structures. The bi-lobed hydrophobic domain appeared to be critical for establishment of these vesicles. Based upon these findings, we predict that chlamydial Inc proteins play an important role in inclusion membrane structure and biogenesis.

## Results

### Formation of Vesicular Structures in HeLa Cells Expressing Inc Proteins

**Figure 1:**
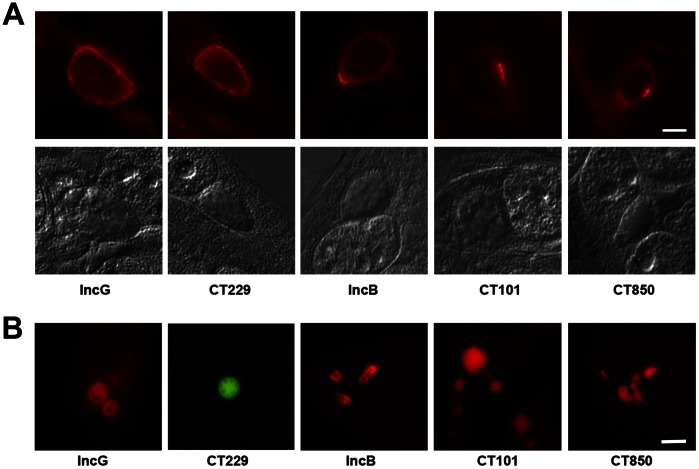
Inclusion membrane localization of specific Incs and corresponding structures when ectopically expressed. A. *C. trachomatis* L2 inclusions at 18 hr post-infection stained for immunofluorescence with specific antibodies to the inclusion membrane proteins IncG, CT229, IncB, CT101, and CT850. IncG and CT229 show circumferential staining patterns while IncB, CT101, and CT850 are enriched in microdomains on the inclusion membrane. Nomarski differential interference contrast images of the same fields are shown for reference. B. The same Incs as above ectopically expressed in HeLa cells as mCherry or GFP fusions. Bar = 10 µm.

*C. trachomatis* inclusion membrane proteins (Incs) display distinct distributions around the inclusion membrane. The initial descriptions of *C. caviae* and *C. trachomatis* Incs were of chlamydial proteins having relatively uniform distribution around the circumference of the inclusion membrane [30]–[32]. Others, such as IncF, are enriched at the point of contact of RBs with the inclusion membrane [32] while another subset of Incs, including IncB, CT101, CT222, CT850 [33] and potentially CT223 [25] are localized in discreet microdomains enriched in cholesterol and host Src-family tyrosine kinases [33] (Fig. 1A).

Selected *C. trachomatis* Incs representative of those expressed circumferentially or in microdomains were expressed in HeLa cells as mCherry or GFP fusions. Each Inc fusion formed distinctive structures in the cytosol (Fig. 1B). These structures appear generally vesicular with rim-like staining patterns although thickness of the vesicle wall was variable. Internal membranous structure was frequently apparent. The Inc vesicles varied in size and occurred singly or multiply. Most often the vesicles were spherical although CT850 tended to form elongated, amorphous structures.

### Ultrastructure of IncB Vesicles

**Figure 2:**
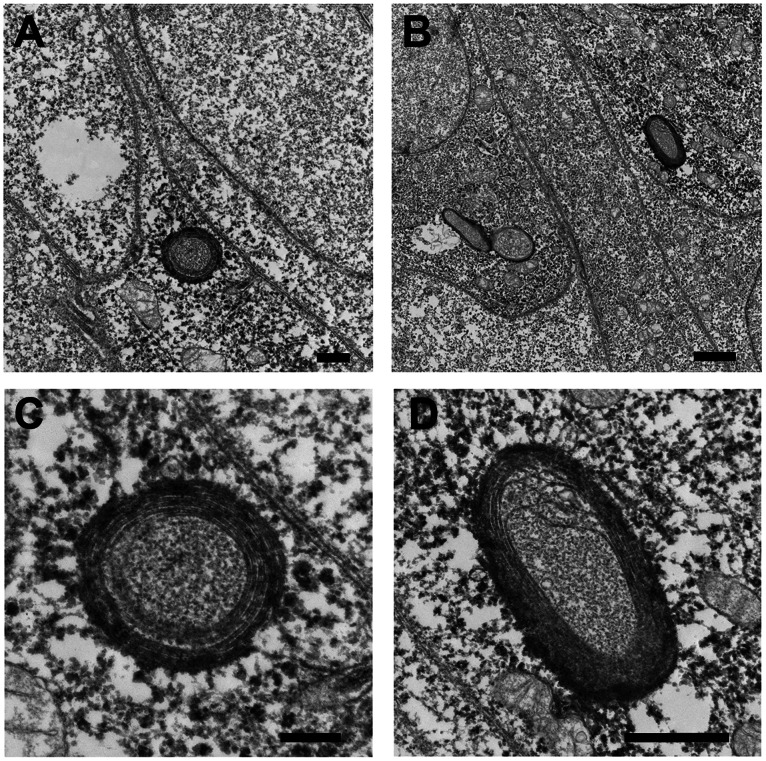
Immunoelectron microscopy of ectopically expressed mCherry-IncB in HeLa cells. A and B. Examples of mCherry-IncB expressed in HeLa cells and immunolabled with an anti-mCherry antibody followed by an HRP-conjugated secondary antibody and developed with a commercial diaminobenzidine substrate. C and D. Higher magnification of the same sections showing internal membrane structure. Bars = 1 µm (A&B); 0.5 µm (C&D).

To confirm the vesicular nature of the structures induced by IncB expression, cells expressing mCherry-IncB were processed for immunoelectron transmission electron microscopy to unambiguously identify the IncB vesicles and examine their ultrastructure (Fig. 2A–D). mCherry-IncB vesicles were spherical or elliptical and displayed a distinct multi-layered membrane appearance. The immunolabeling was localized to the multi-layered membrane but the interior was rich in amorphous material.

### Role of the Hydrophobic Domain in Vesicle Formation

**Figure 3:**
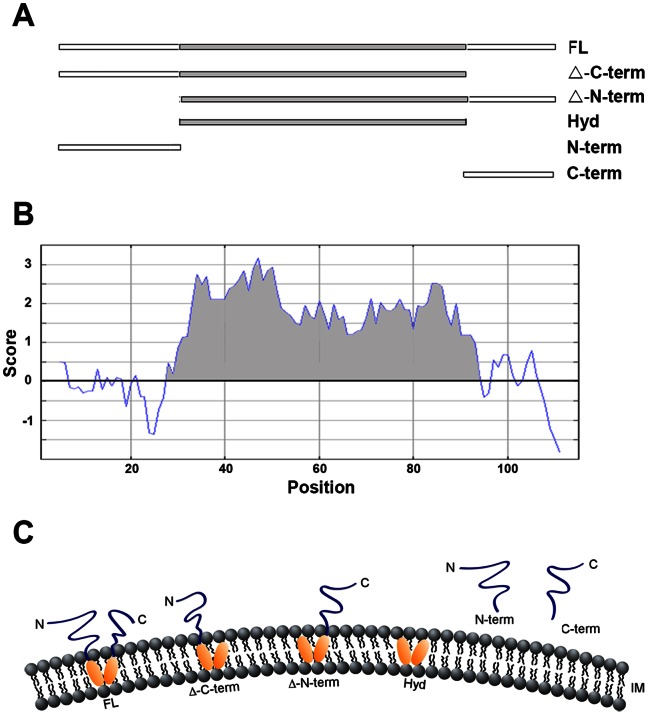
Schematic of IncB fragments expressed as mCherry fusions and predicted membrane topology. A. Line drawing of expressed fragments: FL = Full Length; Δ-C-term = C-terminus deleted; Δ-N-term = N-terminus deleted; Hyd = hydrophobic domain; N-term = N-terminus; C-term = C-terminus. The hydrophobic domain is shaded. B. Kyte-Doolittle hydropathy plot of IncB. The hydrophobic domain is shaded. C. Predicted membrane topology of the various fragments.

**Figure 4:**
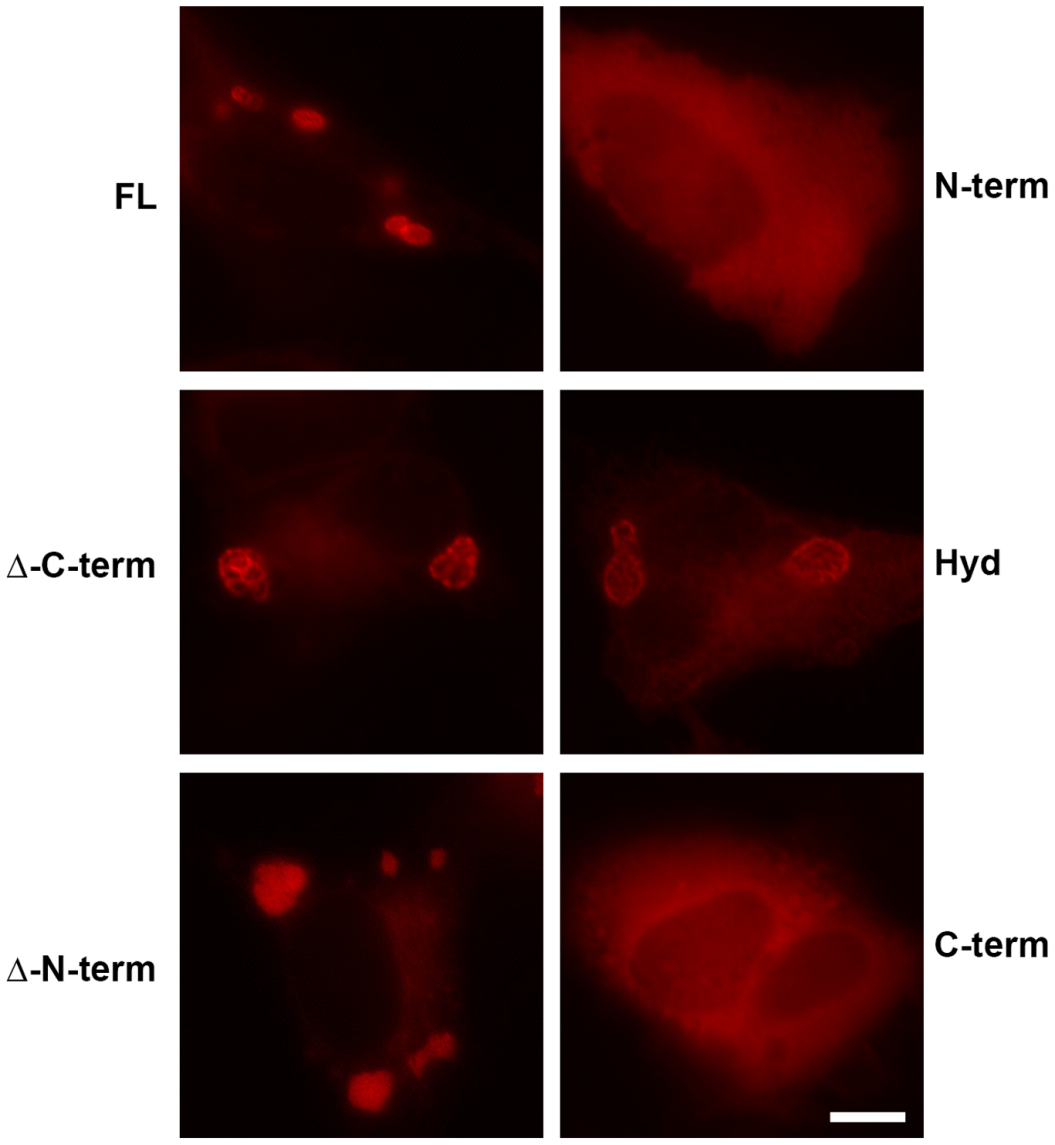
Ectopic expression in HeLa cells of mCherry-IncB fusions depicted in Figure 3. All fragments that contain the hydrophobic domain form visible vesicular structures. Bar = 10 µm.

To explore in greater detail the structures formed by cytosolic Incs, IncB, which contains a centrally located hydrophobic domain, was selected for detailed analysis. Full length IncB as well as the N- or C-terminus with the hydrophobic domain, hydrophobic domain alone, and N- or C-terminus without the hydrophobic domain were expressed as mCherry fusions (Fig. 3). Each of the truncated IncB fragments containing the bi-lobed hydrophobic domain, including the minimal hydrophobic domain alone, formed vesicle-like structures when expressed in HeLa cells (Fig. 4). The small N- and C-terminal fragments lacking the hydrophobic domain remained soluble. Interestingly, the minimal hydrophobic domain appeared to only form vesicles at higher expression levels. At lesser levels of expression, the hydrophobic domain fusion protein seemed to be enriched in the ER. Collectively, the data suggest that the bilobed hydrophobic domain plays an important role in inclusion membrane biogenesis.

### Absence of Host Protein Markers on the IncB Induced Vesicles

**Figure 5:**
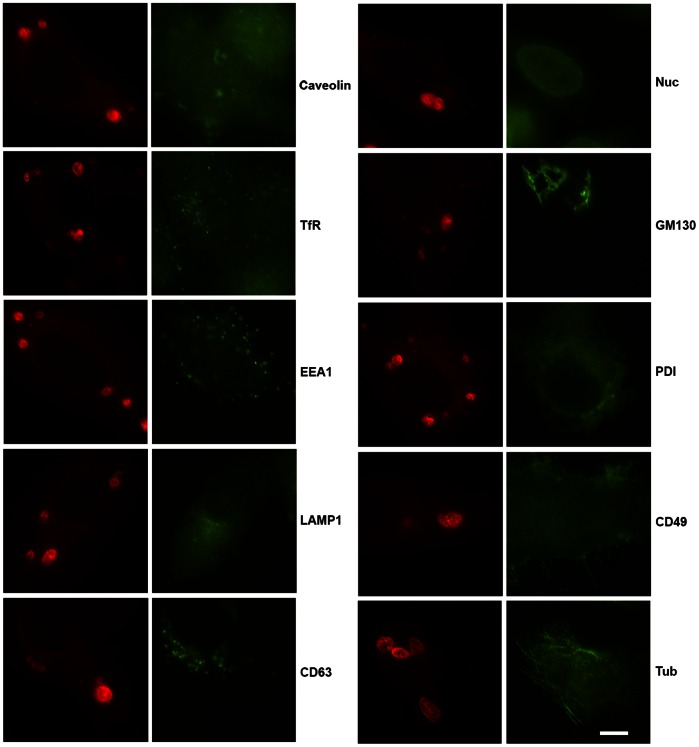
Immunofluorescent staining for various cellular organelles and structures in mCherry-IncB expressing HeLa cells. No colocalization of any markers (green) with IncB induced vesicles (red) was observed. Markers included: Caveolin, transferrin receptor (TfR), early endosomal antigen 1 (EEA1), lysosomal glycoprotein 1 (Lamp1), CD63, nucleolin (Nuc), GM130, protein disulfide isomerase (PDI), CD49, and tubulin (tub). Bar = 10 µm.

**Figure 6:**
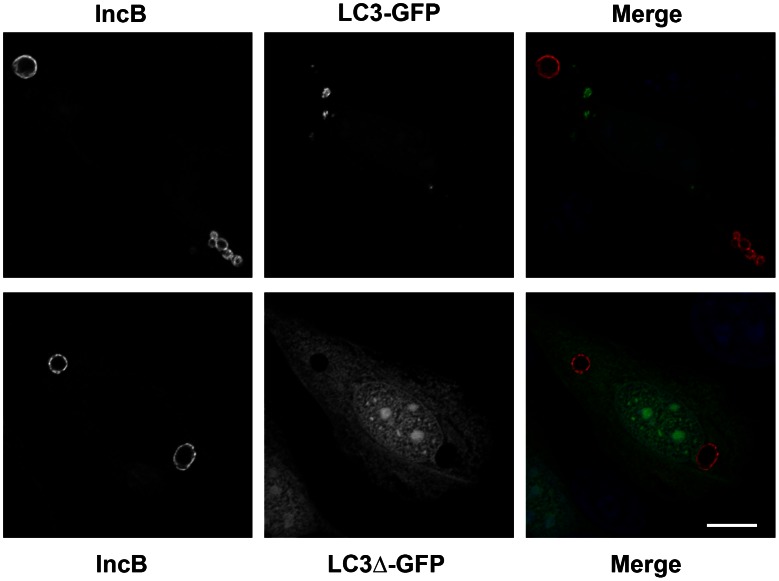
Co-expression of mCherry-IncB and GFP-LC3 as a marker for autophagosomes. GFP -LC3ΔC22, which lacks the C-terminal 22 amino acids, is not correctly processed and does not localize to autophagosomes [34] was used as a negative control. Bar = 10 µm.

To determine whether the IncB-induced vesicles were a result of incorporation into, or trafficking to, host organelles, we screened against a battery of antibodies to various organelles (Fig. 5). None of the antibodies showed localization to the IncB-induced vesicles. Even though ectopically expressed Incs, especially at low expression levels, often appeared in structures reminiscent of the ER, the organized vesicles did not show staining with the ER marker protein disulfide isomerase (PDI). Cells were also co-transfected with GFP-LC3 [34] as a marker for autophagosomes before expression of mCherrry-IncB (Fig. 6). Again, no colocalization was observed. Importantly, markers for lysosomes (Lamp1), autophagosomes (Lamp1 and LC3), and multivesicular bodies (CD63) were negative, indicating that the ectopically expressed protein was not simply delivered to degradative vacuoles.

### Lipid Incorporation by IncB-induced Vesicles

The chlamydial inclusion membrane contains host lipids, such as cholesterol [33], [35], sphingomyelin, at least transiently, as well as neutral lipids [22], diacylgycerol [36], and phosphoinositides [37]. In addition, lipid droplets are translocated across the inclusion membrane by unknown mechanisms where they accumulate in the lumen of the inclusion [38].

**Figure 7:**
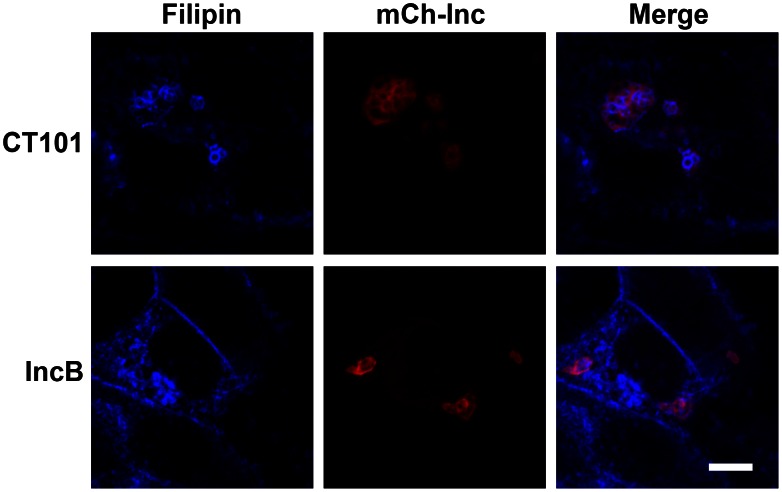
Enrichment of cholesterol in CT101 and IncB induced vesicles. mCherry-CT101 or mCherry-IncB were expressed in HeLa cells and stained with filipin to detect membranes enriched in cholesterol. Bar = 10 µm.

To examine the lipid acquisition by the Inc-induced vesicles, we expressed mCherry-IncB or mCherry-CT101 in HeLa cells and 18 hr after transfection, fixed and stained with filipin (Fig. 7), a polyene antibiotic that preferentially inserts in cholesterol rich membranes [39]. Filipin staining suggests that the CT101 vesicles are enriched in cholesterol but IncB vesicles are not.

**Figure 8:**
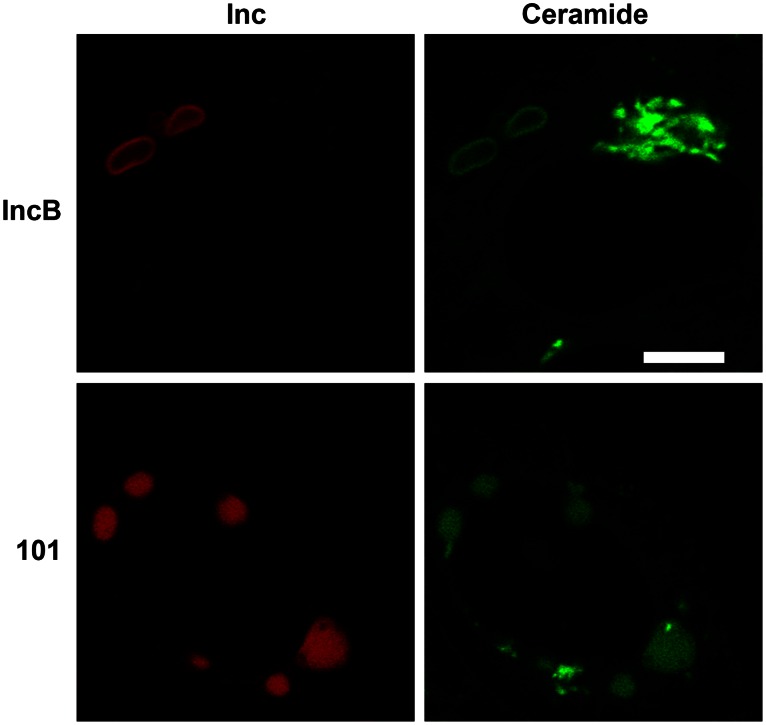
Labeling of CT101 and IncB induced vesicles with C_6_-NBD-ceramide. Cultures were visualized after 5 min of back-exchange [40], [41] to extract plasma membrane localized fluor. Note the distinct rim-like staining patterns of the IncB-induced vesicles as compared to the internal structure of the CT101-induced vesicles. The intense green staining by C_6_-NBD-ceramide represents the Golgi apparatus. Bar = 10 µm.

The trafficking of sphingomyelin to the chlamydial inclusion is one of the hallmarks of chlamydial intracellular growth. Sphingomyelin trafficking is demonstrated by using a fluorescent precursor of sphingomyelin, C_6_-NBD-ceramide, which is modified at the Golgi apparatus by the addition of a phosphocholine headgroup before export [40]. Ceramide itself does not directly label the chlamydial inclusion membrane but requires processing to sphingomyelin before transport to the inclusion [41]. In uninfected cells at 37°C, C_6_-NBD-ceramide labels primarily the Golgi apparatus and no other large organelles [40], [41]. Labeling IncB-mCherry expressing cells with C_6_-NBD-ceramide showed enrichment of fluorescence in both the IncB and CT101 vesicles (Fig. 8). We could not isolate and extract lipid from these structures for analysis but the appearance of fluorescence in the IncB vesicles was evident by 5 min post-temperature shift. This is much more rapid than is observed for trafficking to the chlamydial inclusion [41] and suggests that it is likely ceramide rather than sphingomyelin that is accumulated within these vesicles.

**Figure 9:**
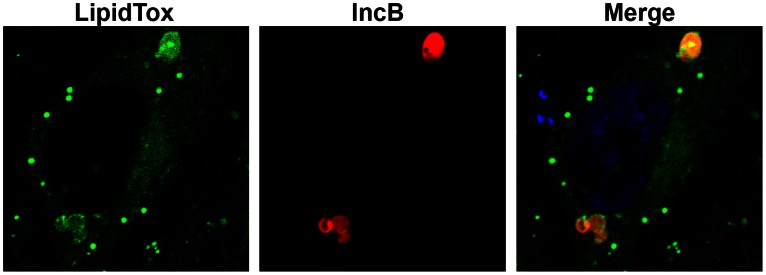
Enrichment of neutral lipids in IncB induced vesicles. mCherry-IncB was expressed in HeLa cells and stained with LipidTox to detect neutral lipids. Numerous lipid droplets (green) are visible throughout the cell as is enrichment at the IncB vesicles. Bar = 10 µm.

Recruitment of neutral lipids was analysed by observing IncB tranfected cells stained with LipidTOX (Fig. 9). LipidTOX stained lipid droplets within the cytosol of transfected cells but also stained the IncB vesicles. Occasionally, lipid droplets were observed within the IncB vesicles. Although the composition of the IncB vesicles, and the chlamydial inclusion membrane, are unknown it appears that both structures contain at least cholesterol, ceramide, and unknown neutral lipids are enriched in these structures. The enrichment of specific lipid species suggests that the presence of chlamydial inclusion membrane proteins may influence the composition and structure of the inclusion membrane.

### Co-localization of Ectopically Expressed Incs

**Figure 10:**
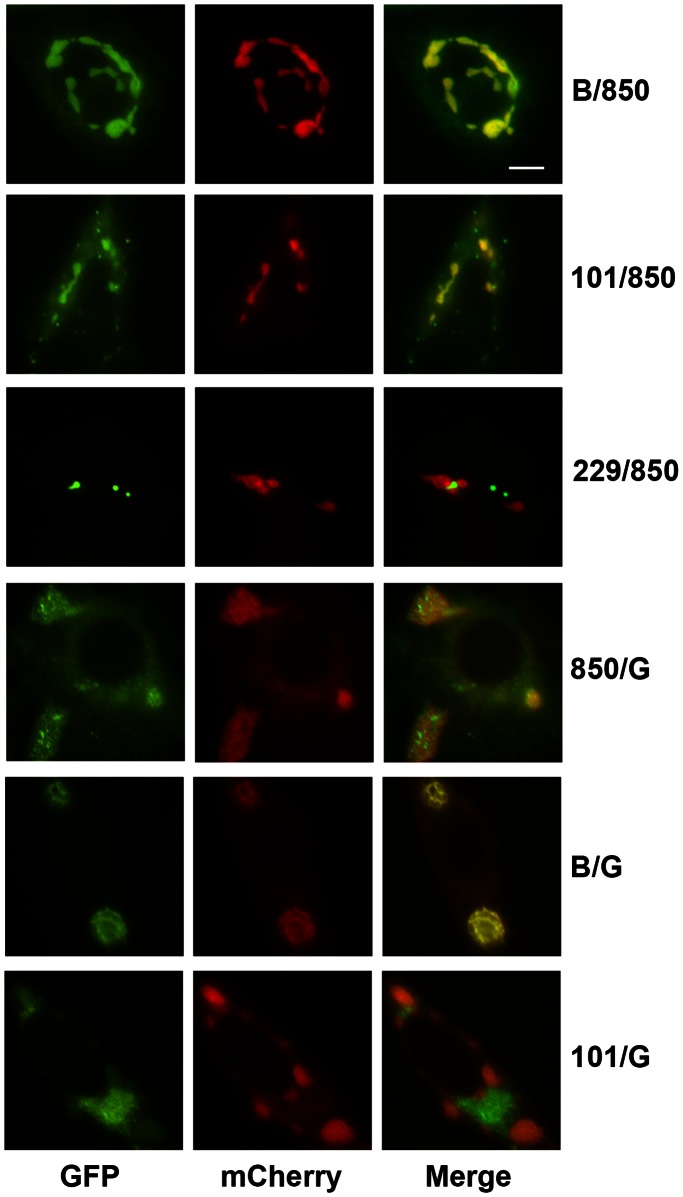
Ectopic expression of Inc pairs. Incs were co-expressed as mCherry or GFP fusions to determine recruitment to the same vesicles. Inc pairs are identified as GFP (left panel)/mCherry (middle panel). Bar = 10 µm.

We also co-expressed mCherry- and GFP-Inc pairs ectopically in HeLa cells (Fig. 10). Virtually all possible combinations of co-localization or segregation were observed for the pairs. For example, GFP-IncB and mCherry-850 entirely co-localized in large amorphous structures similar to that of mCherry-850 alone. GFP-101 and mCherry-850 largely co-localized but small vesicular structures comprised of GFP-101 alone are evident throughout the cytoplasm and not incorporated into the major, large aggregate. GFP-229, however, did not localize at all with mCherry-850 suggesting that the lipid environments created by ectopic Inc expression may not be compatible with insertion of all Incs.

## Discussion

Like many obligate intracellular parasites, chlamydiae possess a relatively reduced genome presumably due to their occupancy of a nutrient rich environment from which they can scavenge many essential nutrients and precursors. Although chlamydiae encode a genome of only 1.04 Mbp [42], they commit approximately 4 percent of their coding capacity to a family of proteins that modify the inclusion membrane. Inclusion membrane proteins are largely identified based upon a bi-lobed hydrophobic domain of 40 or more amino acids in length; sufficient to span a typical membrane bilayer twice. Estimates of the number of Incs encoded by *C. trachomatis* exceed 50 [11], [23], [25]–[28]. Other chlamydial species are predicted to encode an even greater number. Here we find that ectopically expressed Incs contribute to the formation of unique membranous vesicles whose structure is determined at least in part by the specific Inc expressed. Remarkably, chlamydial inclusion membrane proteins expressed ectopically in eukaryotic cells appear to insert into available host membrane to effectively hijack cellular membranes to create an anomalous vesicular structure. Efforts to characterize these aberrant vesicles by localization with markers for various cellular organelles were uniformly negative. One plausible explanation might be the the Incs may displace endogenous cellular membrane proteins. Such a mechanism may help explain the dearth of cellular markers on the inclusion membrane.

Despite the large number of Incs, there is little sequence similarity between them nor clues as to function based upon bioinformatic analyses. A majority of *C. trachomatis* Incs are synthesized early during infection and thus are believed to play an important role in establishment of an appropriate replicative niche for chlamydiae [23]. There is, however, a notable lack of identified function for individual Incs. IncA was first identified in *C. caviae* [30], [31] although its one known function is observed only in *C. trachomatis*. IncA has been implicated in the characteristic homotypic fusion of inclusions in cells infected with multiple *C. trachomatis* EBs [43]–[45]. It has been proposed that this role is determined by structural similarities of IncA to SNARE-like motifs [46] which are characteristic of eukaryotic membrane proteins that regulate vesicle fusion. Homotypic vesicle fusion is not, however, an essential function for *C. trachomatis* replication or virulence since microinjected antibodies to IncA inhibit fusion but do not inhibit development [43]. Additionally, IncA is also poorly conserved between chlamydial species and *C. trachomatis* clinical isolates deficient in IncA have been identified [44].

Interactions of other Inc proteins with various eukaryotic components have been reported although most of these associations are not shared throughout the genus. The host adaptor protein, 14-3-3β is recruited to *C. trachomatis* IncG [47] where it has been proposed to sequester the pro-apoptotic protein, BAD, to protect the infected cell from apoptosis [48]. Neither expression of IncG, nor recruitment of 14-3-3β, is observed, however, on the inclusion membranes of *C. caviae* or *C. pneumoniae* [47]. The *C. trachomatis* Inc, CT229, recruits Rab4 to the inclusion membrane [49]. Rabs constitute a family of small GTPases that are important regulators of vesicle fusion in eukaryotic cells. Rab proteins are recruited in unique combinations to different chlamydial species. For example, Rab1, Rab4, Rab11, and Rab14 are recruited to *C. trachomatis, C. muridarum,* and *C. pneumoniae* inclusions but Rab 6 is specific to *C. trachomatis* and Rab10 is specific to *C. muridarum*, and *C. pneumoniae* [50], [51]. The *C. pneumoniae* Inc, Cpn0585, interacts with Rab1, Rab10 and Rab11, but not Rab4 or Rab6 [52]. Recently, *C. trachomatis* IncD has been implicated in the recruitment of CERT, a ceramide endoplasmic reticulum transferase, which functions in delivery of ceramide from the ER to the Golgi apparatus, and proposed to function in the delivery of sphingomyelin to chlamydiae [53], [54]. IncD, however, is not conserved among chlamydial species [26], [29] although sphingomyelin trafficking is common to all [41], [55], [56].

Although little structural information is available regarding chlamydial Incs beyond the secondary structural predictions, they are regarded as intrinsic membrane proteins with a hairpin structure such that the N- and C-terminal hydrophilic domains are exposed to the cytosol [18], [26]. The limited data obtained from microinjection of antibodies to these domains are consistent with this topology [18], [43], [57]. Intrinsic membrane proteins can potentially alter membrane curvature by changing the lipid composition of one of the bilayers. Alternatively, transmembrane proteins with a conical shape may induce curvature depending upon their shape [58]–[60]. The inclusion membrane proteins, which share a bi-lobed hydrophobic secondary structure without primary amino acid sequence similarity, could therefore exert distinct effects on local membrane structure. Indeed, specific Incs expressed in HeLa cells are organized in unique membranous structures that exhibit distinct shapes and display different affinities for diverse lipid probes thus inclusion membrane proteins could potentially influence inclusion membrane structure and function through either or both mechanisms. Although the membranous structures induced here by expressed Incs appeared multilamellar and in various forms, it should be noted that the Incs are naturally secreted via a type III secretion system from within a parasite-occupied parasitophorous vacuole. Incs also do not appear in nature individually but are expressed as family of proteins which, in composite, may contribute collectively to inclusion membrane structure and function. It is therefore unsurprising that these vesicles do not entirely resemble a native chlamydial inclusion. The data suggest, however, that chlamydial Incs can influence membrane architecture. A more detailed analysis of Inc impact on membrane composition and structure will likely necessitate a biophysical approach using artificial membranes of defined compositions and selected peptide domains of specific Incs individually or in composite.

The cytosolically exposed domains of Incs are susceptible to proteolytic cleavage and processing for antigen presentation at the cell surface [25], [61], [62]. One possibility is that the large number of Inc proteins might permit, collectively, a substantial structural role without reliance upon an abundance of individual Incs that could trigger immune surveillance mechanisms. Alternatively, the diversity in Incs may communally provide for the appropriate lipid composition of the inclusion membrane. Certainly, local enrichment of specific Incs is known to influence lipid composition in specific domains of the inclusion membrane [33]. The large number of different Incs, lack of conservation between species, and apparent lack of genus-wide conserved functions suggests that a structural role in inclusion membrane organization may be among the critical functions of this unique family of chlamydial proteins.

## Materials and Methods

### Organism and Cell Culture

Chlamydia trachomatis serovar L2 (LGV 434) was propagated in HeLa 229 cells and purified by Renografin density gradient centrifugation as previously described [63].

### Plasmids and Transfections

mCherry was fused to the N-terminus of full length Inc proteins and the IncB fragments depicted in Fig. 3 using the XhoI and EcoRI sites in pmCherry-C1 (Clontech, Mountain View, CA). EGFP fusions to the N-terminus were similarly constructed using pEGFP- C1 (Clontech). Each Inc gene was amplified from *C. trachomatis* serovar L2 DNA using forward primers (IDT, Coralville, IA) that incorporated an XhoI or BamH1site (IncB: CCC CTC GAG GGA TGG TTC ATT CTG TAT ACA ATT CAT TG; Inc101: CCC CTC GAG GGA TGA TCT CCA TGA TTC CAA GG; Inc850: CCC CTC GAG GGA TGG GAT TCG GAA CTG TGA GAG G; IncG: CCC GGA TCC TTA GAA GGA GCG TGA TCG AGA ACG GC) and reverse primers (IDT) that incorporated an EcoRI site (IncB: CCC GAA TTC CTA TTC TTG AGG TTT TGT TGG GCT G; Inc101: CCC GAA TTC TCA GTA ATA ATA AAC AGA ATA TTT TGA TTT TAA C; Inc850: CCC GAA TTC TTA CCG ATT CTG GTT GTG AAG TAC TAA C; IncG: CCC FAA TTC GAT GAT CTG TTG TGA CAA AGT C). EGFP-CT229 was previously described [64].

These plasmids were used to transfect HeLa cells on glass cover slips in 24 well plates using Lipofectamine reagents (Invitrogen) according to the manufacturer’s instructions.

### Immunofluorescent Microscopy

HeLa cells were plated on glass coverslips in 24-well plates (Corning). *C. trachomatis* L2 infections were performed in Hanks Balanced Salt Solution (HBSS, Invitrogen/Gibco, Carlsbad, CA). After 1 hr incubation at 37°C, the medium was removed and replaced with pre-warmed RPMI-1640+10% FBS. The cultures were incubated an additional 18 hr before fixation in methanol and immunofluorescent staining with anti-Inc antibodies as previously described [33].

For co-localization of host cell markers, HeLa cells on 12 mm glass coverslips were transfected with mCherry-IncB and 28 hr later fixed with methanol for 20 min at room termperature. Antibodies used were caveolin 1, early endosomal antigen 1, LAMP 1, CD63, paxillin, Nucleoporin, CD49 (BD Transduction Labs, San Jose, CA), protein disulfide isomerase 1 (Novus Biologicals, Littleton, CO), β-tubulin (Cell Signaling, Danvers, MA) and GM130 (Epitomics, Burlingame, CA). Secondary antibodies were anti-mouse IgG DyLight 488 or anti-rabbit IgG DyLight 488 (Jackson ImmunoResearch Labs, West Grove, PA).

The plasmids expressing GFP-LC3 and GFP-LC3ΔC22 [34] were kind gifts of Dr. T. Yoshimori.

### Transmission Electron Microscopy

HeLa cells were grown on Thermanox coverslips (Nunc) and transfected with mCherry-IncB for 24 hrs. Cells were rinsed twice with HBSS and fixed with periodate-lysine-paraformaldehyde (PLP fixative: 75 mM lysine, 37 mM sodium phosphate, 10 mM sodium periodate, 2% paraformaldehyde) plus 0.25% glutaraldehyde for 2 hrs at room temperature. Samples were rinsed twice with PBS, permeabilized with 0.01% saponin in PBS for 5 minutes at room temperature, and incubated with rabbit anti-mCherry (Biovision, Milpitas, CA) in 0.01% saponin/PBS overnight at room temperature. The cells were rinsed twice with PBS, incubated with peroxidase conjugated F(ab′)_2_ donkey anti-rabbit IgG (Jackson Immunoresearch) in 0.01% saponin/PBS for 1 hr at room temperature and rinsed three times with PBS. The samples were then fixed for 1 hr with 1.5% glutaraldehyde in 0.1 M sodium-cacodylate pH 7.4 plus 5% sucrose and rinsed three times in 50 mM Tris-HCl pH 7.4 plus 7.5% sucrose. The reactions were developed using Immunopure Metal Enhanced DAB reagent (Pierce Chemical, Rockford, IL). Samples were rinsed three times with 50 mM Tris-HCl with 7.5% sucrose and fixed overnight at 4 °C in 2.5% glutaraldehyde in 100 mM sodium cacodylate buffer, pH 7. Cells were post-fixed two times in 1.0% osmium tetroxide reduced with 1% potassium ferricyanide using a Pelco Biowave laboratory microwave system (Ted Pella, Inc., Redding, CA) at 250 W (2 min on, 2 min off, 2 min on) under 20- in. Hg Vacuum. The cells were later washed with water and dehydrated in graded ethanol series for 45 seconds each in a microwave at 250 W. The cultures were embedded in Spurr’s resin and sectioned with a UC6 ultramicrotome (Leica Microsystems). Micrographs were acquired using a Tecnai Spirit TEM (FEI, Hillsboro, OR) at 120 kV and recorded on 2048×2048 pixel Gatan CCD camera.

### Fluorescent Lipid Markers

C_6_-NBD-ceramide labeling of mCherry-IncB and mCherry-CT101 expressing HeLa cells was performed as previously described [41] except the back-exchange was limited to 5 min at 37°C. Cholesterol was visualized using 50 µg/ml filipin (Sigma) as previously described [35]. LipidTox (Molecular Probes) staining was performed according to the manufacturer’s recommendations.

Images were acquired on a Nikon Eclipse 80 i microscope using a 60×1.4 NA oil immersion objective (Nikon, USA) or a Zeiss LSM 510 Meta laser confocal scanning microscope using a 63×, 1.4 NA oil objective (Carl Zeiss MicroImaging, Inc., Maple Grove, MN).
